# Through Wall Radar Classification of Human Micro-Doppler Using Singular Value Decomposition Analysis

**DOI:** 10.3390/s16091401

**Published:** 2016-08-31

**Authors:** Matthew Ritchie, Matthew Ash, Qingchao Chen, Kevin Chetty

**Affiliations:** 1 Department of Electronic and Electrical Engineering, University College London, London, WC1E 7JE, UK; m.ash@ucl.ac.uk; 2 Department of Security and Crime Science, University College London, London, WC1H 9EZ, UK; qingchao.chen.13@ucl.ac.uk (Q.C.); k.chetty@ucl.ac.uk (K.C.)

**Keywords:** micro-Doppler, FMCW radar, through-the-wall, classification

## Abstract

The ability to detect the presence as well as classify the activities of individuals behind visually obscuring structures is of significant benefit to police, security and emergency services in many situations. This paper presents the analysis from a series of experimental results generated using a through-the-wall (TTW) Frequency Modulated Continuous Wave (FMCW) C-Band radar system named *Soprano*. The objective of this analysis was to classify whether an individual was carrying an item in both hands or not using micro-Doppler information from a FMCW sensor. The radar was deployed at a standoff distance, of approximately 0.5 m, outside a residential building and used to detect multiple people walking within a room. Through the application of digital filtering, it was shown that significant suppression of the primary wall reflection is possible, significantly enhancing the target signal to clutter ratio. Singular Value Decomposition (SVD) signal processing techniques were then applied to the micro-Doppler signatures from different individuals. Features from the SVD information have been used to classify whether the person was carrying an item or walking free handed. Excellent performance of the classifier was achieved in this challenging scenario with accuracies up to 94%, suggesting that future through wall radar sensors may have the ability to reliably recognize many different types of activities in TTW scenarios using these techniques.

## 1. Introduction

There is a significant body of academic research which has attempted to apply radar detection and imaging techniques to scenarios that consist of an obscuring wall barrier, which is summarized effectively within [1,2,3]. Information obtained about the structural layout of a room, if a person is present or how many individuals are present is vital for police, military or civilian search and rescue teams [4]. In the domain of defence and security, a key challenge that exists is the ability not only to sense the presence of individuals within a room but to also classify their actions. This can be accomplished using a multitude of different techniques and types of sensors, each with their own advantages and disadvantages. Invasive positioning of optical sensors allows for observations to be made of a room, but requires physically penetrating the walls or the opening of the closed room which may be impractical in a short time frame or cause disruption that would alert the individuals within the room. The advantage of TTW radar is that it can sense targets within a room at a standoff distance without the need of physically placing the sensor within the room.

The challenge of TTW radar imagery has been researched using a combination of both innovative signal processing and hardware techniques. Hardware innovations in this area include active MIMO array systems [5] providing cross range information, ultra-wide band radars [6,7] for high range resolution, Synthetic Aperture Radar (SAR) imaging techniques [8] and passive WiFi radar [9]. The practical implications of using a MIMO radar for TTW imaging is discussed in [7]. This system was capable of real-time processing and high range and cross range resolution, although it was bulky in its design compared to other man‑portable solutions. These hardware innovations have been successful in gathering information within through wall scenarios, but often come with increased complexity and cost.

Previously developed signal processing techniques that have also been applied to TTW imaging include the MUSIC algorithm [10], compressed sensing [11], image alignment procedures [12] and spatial filtering [13]. The focus of these techniques is often on the maximization the signal-to-noise ratio (SNR) of the desired target or removal of the significant primary wall reflection. TTW human micro-Doppler signatures have been both simulated and analysed previously within [14,15,16,17]. In comparison with those publications, this article focuses on the application of classification of movements on real data obtained in a through wall configuration using human micro-Doppler signatures. The most significant effect of a TTW scenario on the detected signatures is the attenuation of the signals obtained due to the significant loss of signal when propagating through the wall. Other important effects to take into account include multipath and shadowing effects from other clutter items within the scene. Multipath issues are one of the most challenging problems and can result in missed detections or incorrect estimates of the number of targets. All of these problems contribute to a very challenging situation which is yet to be effectively solved.

The hardware system used for this research was a Frequency Modulated Continuous Wave (FMCW) radar which benefits from no minimum blind range, relative low-cost, good resistance to interference, and simple architecture suited to a compact design [18]. This type of radar system has been researched for many decades, with numerous variations on the engineering techniques applied to maximize the information obtained and reduce the effects of clutter and the reflections from the wall. Previous FMCW radar systems that have been used in TTW experiments include work published by ONERA [19,20] showing a UWB radar (1–5 GHz) measuring a person walking inside a residential building as well as evaluating the measured attenuations with respect to range and frequency. A specific application of an FMCW system was in the measurement of a human breathing through wall [21,22] achieved using an S-Band Self Injection Locked (SIL) architecture. The wall used for this case was, however, only a plywood barrier in comparison to a standard brick wall with air gaps.

The micro-Doppler effect is generated by the rotation or vibration of components of a target and presents itself as modulations on top of the main Doppler shift generated by the bulk translational motion of a target [23]. In the case of human micro-Doppler, the motion of the arms, legs and torso all contribute to a unique signature modulated on the main Doppler velocity generated by an individual moving forward. The study of human micro-Doppler is an active area of interest in radar research, with the main objective often being the extraction of additional information within the micro-Doppler signature for the radars’ advantage. Well defined models for human motion have been developed by V. Chen and applied to real data [23,24,25,26], these can accurately produce expected micro-Doppler contributions from different types of motions. In recent work it has been shown that optical sensors can also be used to develop human micro-Doppler modelling capabilities further through the use of a simple low cost optical sensor [27].

The additional information provided by the human micro-Doppler can be used to classify the types of motions individuals are making, from walking to boxing, or to classify different targets, for example animals, humans or vehicles [28], or for Automatic Target Recognition (ATR) [29]. Youngwook and Hao [28], showed seven different human motions could be successfully classified by appling a Support Vector Machine technique, although this was completed in a non-TTW scenario. If the information is maintained within the signal after propagating through a wall, then the same processing should, in theory, be possible, although with potential reductions in performance due to lower SNR and the additional multipath components.

The analysis of human micro-Doppler has also been performed using multistatic radar sensors, where a comparison was made between a simple model for human motion and the real multistatic micro-Doppler data [30]. In a multistatic scenario, classification methodologies have been applied in order to differentiate individuals walking free handed or while carrying a rifle‑like object [31,32]. This analysis used either empirical manually extracted features from the micro-Doppler signatures or an automated Singular Value Decomposition (SVD) feature extraction. The techniques applied within this work follow on from the SVD processing methodologies applied to the multistatic data [31], but are utilized in a TTW scenario, using a different radar sensor, with a different sensor architecture and alternative classification techniques. In the non-TTW configuration, classification results achieved by SVD processing were shown to have success rates of up to 95% with 25% training samples. SVD processing has previously been applied to through wall radar analysis but the focus has not been on using this information as part of classification processing. It was shown in [33] that SVD can be used as part of the detection process when using a TTW radar, although this work focused solely on the detection of a target using the SVD information, not the classification of the actions of a human target. SVD processing was also used for TTW radar in [34], but this was to mitigate clutter, not for detection or classification.

The rest of this paper is organized as follows. Section 2 presents the radar system and the experimental setup used for the data collection process. Section 3 explains the theory behind the SVD processing and the classifiers that have been applied to the data. Section 4 shows the results of these signal processing algorithms and compares the classification accuracy from the different cases measured. Finally, Section 5 concludes the paper with a summary of results that have been presented and defines potential future expansions on this research.

## 2. Radar System and Measurement Setup

**Figure 1:**
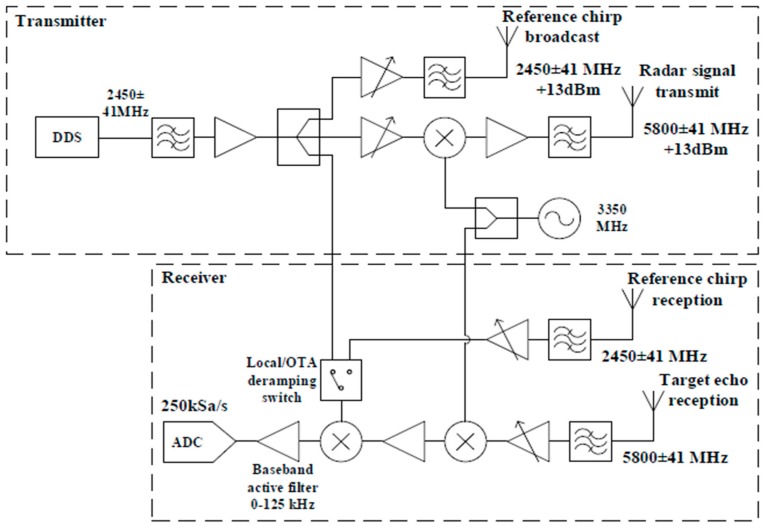
Soprano radar block design.

The Soprano radar system was developed at University College London (UCL) in 2014. The radar uses a FMCW architecture operating at C-band with a central frequency of 5.8 GHz. This system was manufactured using commercial off‑the‑shelf (COTS) surface mount components on an in-house designed PCB [35,36]. A basic block diagram of the system design can be seen in Figure 1 along with the RF parameters in Table 1. The Direct Digital Synthesiser (DDS) chip used within the system is capable of a bandwidth of 200 MHz but is limited to 83.5 MHz to meet ISM bandwidth regulations in the UK. The system is capable of operating in monostatic or bistatic mode, using an over-the-air deramping (OTAD) technique unique to this radar [35]. However, for all the results presented here the system was only operated in a monostatic configuration.

Digitization of the output baseband signal was performed using a USB external audio card controlled using Matlab (Natick, MA, USA) on a standard laptop. This is possible due to the low output frequency of the deramped FMCW signal. An external sound card is a good candidate for an Analogue to Digital Converter (ADC) in this case as it is low cost, small, easy to setup, and the data can be read by most programming languages. The sound card has 24-bit dynamic range and is capable of measuring signals up to 98 kHz. As the exact start of a chirp period was challenging to sample, with the slow ADC used, a second channel of the audio card was used to sample the output from a pulse stretching device which elongates the instance of the beginning of the chirp to a duration that can be sampled by the audio card. Through the use of interpolation, it was then possible to break down the long string of data samples into individual chirps which were first windowed using a hanning window to reduce range side-lobes and then filtered using a high-pass Finite Impulse Response (FIR) filter across multiple chirps of data. The FIR filter was applied as part of the Moving Target Identification (MTI) processing on the data to enhance only moving targets within the scene. This data was then Fourier transformed in order to obtain range-time profiles which could be used to identify where a moving target was within the scene.

**Figure 2:**
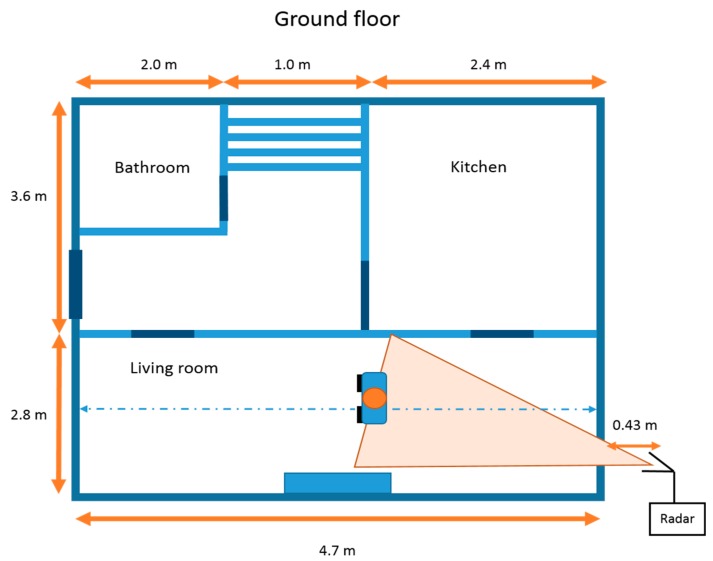
Geometry of the experimental setup showing individual walking up and down centrally within living room space and radar deployed at 43 cm distance.

**Figure 3:**
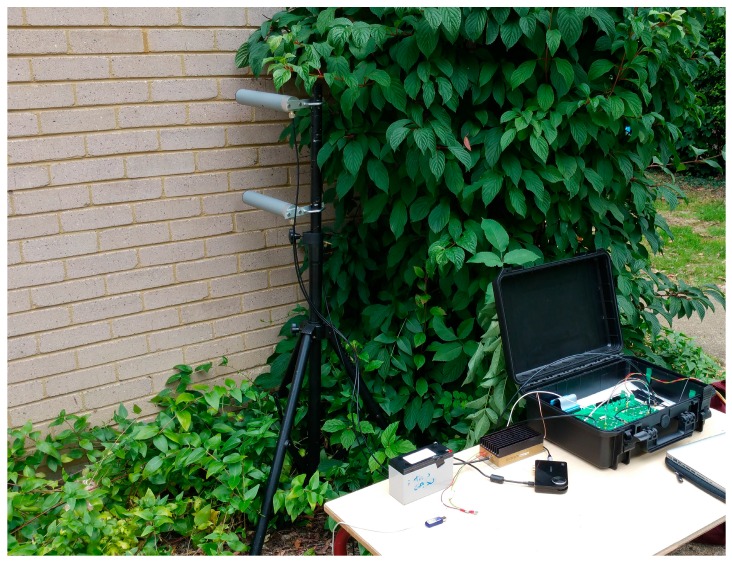
Image of Soprano radar deployed outside of building.

The experiments were performed at a UCL test site in Shenley, UK. The building used was a residential house with a brick wall that was approximately 33 cm thick, with a central air gap. The radar system was setup outside of the property with the antennas at a distance of 43 cm from the exterior wall. The room had a floor to ceiling height of approximately 2.4 m and no furniture was present within the area of interest. This represented a very challenging real world scenario for a through wall radar; features such as the chimney-breast, electrical wires, and pipes within the walls all contributed to a complex cluttered environment. The geometry of the building and radar location can be seen in Figure 2, along with an image of the Soprano radar deployment in Figure 3.

## 3. Theory

The application of SVD allows for the decomposition of the dimensionality of a matrix into fundamental components of the original signal, comparable to a form of compressed sensing. The assumptions made when performing SVD processing are that the signal of interest can be deconstructed into a linear combination of a few key components from a defined dictionary [37]. This methodology is applicable to micro-Doppler classification, as the spectrogram matrix is a suitable candidate for sparse representation due to only a few key features containing a significant proportion of the vital information. SVD signal processing enables a sparse representation of this data through subspace base analysis. The SVD as well as eigenvalue decomposition (EVD) processing techniques have been previously used for subspace estimation, despite their computationally demanding algorithms. Modifications to these techniques include sliding window adaptive implementations [38] and a K-SVD representation [39] of the model. For the analysis within this paper, a simple representation of the SVD model is used to analyse the real data produced by the radar in order to extract features to input into a classifier. The advantage of the SVD processing technique is that physical empirical features from the micro-Doppler signature, such as period and bandwidth, do not have to be manually extracted prior to classification, making the processing automated and easily repeatable.

This theorem is based on the concept that any matrix **A** is defined as,

(1) $$A\in R^{m\times n}$$

When matrix ***A*** has rank of one, the matrix can be represented by,

(2) $$A=u\sigma \upsilon^{T}$$

where *u* ∈ **R**^m^, *υ* ∈ **R**^n^, and *σ* > 0. This can be expanded to when matrix **A** has an arbitrary rank of *r*, where the matrix can be represented by a sum or rank one matrices.

(3) $$A=\overset{r}{\underset{i=1}{\sum }}\sigma_{i}u_{i}\upsilon_{i}^{T}$$

where *r* is the rank of the matrix, *u_i_* and *υ_i_* values are all mutually orthogonal and the *σ_i_* values are all positive and labelled as singular values of the matrix **A**.

It is the matrices *υ* and *u* that have been evaluated for each of the micro-Doppler datasets that are available. The *σ* values are only scaling values for the unity matrices *u* and *v*, and are, hence, not used as part of the classification process. Features from within these matrices are extracted in order to perform a classification process.

The classifiers that have been applied to the data are Discriminant Analysis (DA), which is a form of a Bayesian classifier, and Random Forest (RF) classifier types. The DA classifier is based on the assumption that the features’ vectors inputs are both Gaussian distributed and statistically independent. The two categories of DA classifiers that have been applied are linear and quadratic; for the linear case, the model has the same covariance matrix for each class, only the means vary, while for the quadratic case, both means and covariance of each class vary [40,41]. During the training process the mean and variance of these feature vectors are estimated as part of the classification process. Then, the posterior probability of each sample under test belonging to each class can be evaluated in order to assign the sample to a given class which gives the highest probability. This is achieved by minimizing the expected classification cost, which is defined in [42] as:

(4) $$\hat{y}=\underset{y=1,\ldots .,K}{\arg\;\min}\overset{K}{\underset{k=1}{\sum }}\hat{P}\left(k|x\right)C\left(y|k\right)$$

where *K* is the number of classes, y is the predicted classification, $\hat{P}\left(k|x\right)$ is the posterior probability of class k for observation x and C(y|k) is the cost of classifying an observation as y when its true class is k. The posterior probability is the product of the prior probability $\hat{P}\left(k\right)$, which has been assumed to be uniform across all classes within this work, and the multivariate normal density of the features which is defined as:

(5) $$P\left(x|k\right)=\frac{1}{2\pi \left|\Sigma_{k}\right|}exp\left(-\frac{1}{2}{\left(x-\mu_{k}\right)}^{T}\Sigma_{k}^{-1}\left(x-\mu_{k}\right)\right)$$

With *μ_k_* representing the mean and Σ*_k_* representing the covariance. Using Equation (5) with the prior probability, it is possible to evaluate the posterior probability that observation x is from class k by using the Bayes rule shown below:

(6) $$\hat{P}\left(k|x\right)=\frac{P\left(x|k\right)P\left(k\right)}{P\left(x\right)}$$

This is a component of Equation (4) that required minimization as part of the classification decision-making process.

**Figure 4:**
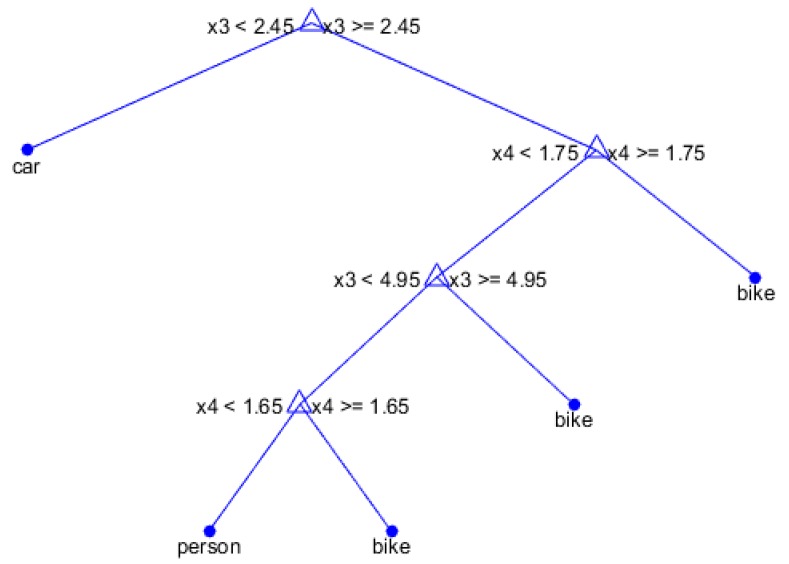
Random Forest Tree example.

The second type of classifier that was applied was the RF process [43,44], which is a supervised machine learning algorithm that uses a series of binary decisions configured in a tree form which allow for the categorization of a given input to a series of classes. A decision tree is formed by considering all possible split combinations which are available from all feature samples, and then the optimum splits are defined using an optimization criterion. A random component of the decision tree process is introduced by utilizing a random subset of the given data to construct a series of decision trees in an iterative way. The purpose of this randomization is to minimize the error in the classification process by testing the numerous different generated decision trees. The error that is being minimized is the Gini Diversity Index (GDI), which is defined as:

(7) $$g\left(n\right)=1-\underset{k}{\sum }p^{2}\left(k\right)$$

where g is the GDI, k is the possible classes and p is the fraction of classes with the correct classification that have reached a given node within a RF decision tree. An example RF tree using a three class problem (car, person, bike) and four features (x1, x2, x3, x4) can be seen in Figure 4. At each fork of the tree a decision is made on each sample based on a greater than or less than aspect of some of the selected features. At the leaf end of each branch the decision for each sample is indicated by the class. For example, if feature x3 is < 2.45 the sample will immediately be classified as a car by the first fork of the tree.

## 4. Data Analysis and Classification

**Figure 5:**
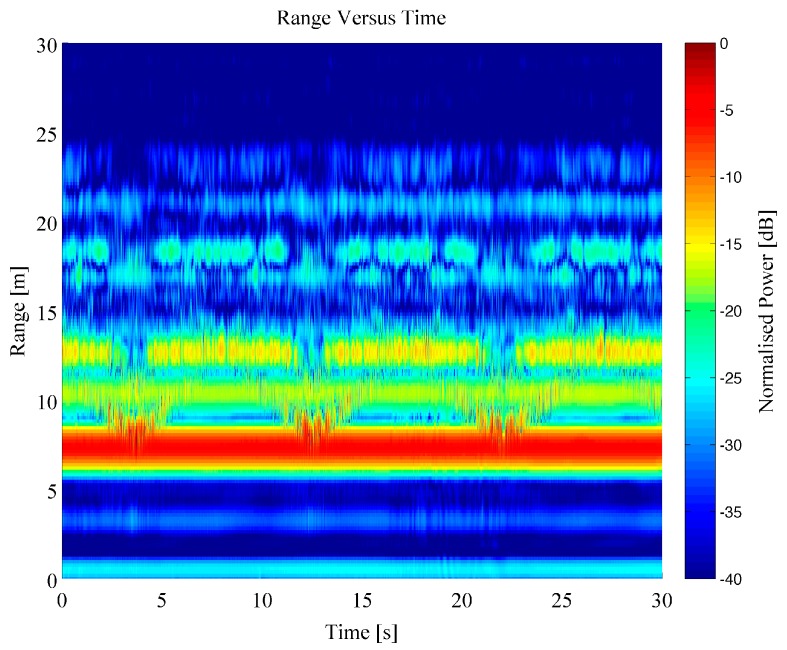
Range Time Intensity (RTI) plot of individual walking with free hands measured through the wall.

**Figure 6:**
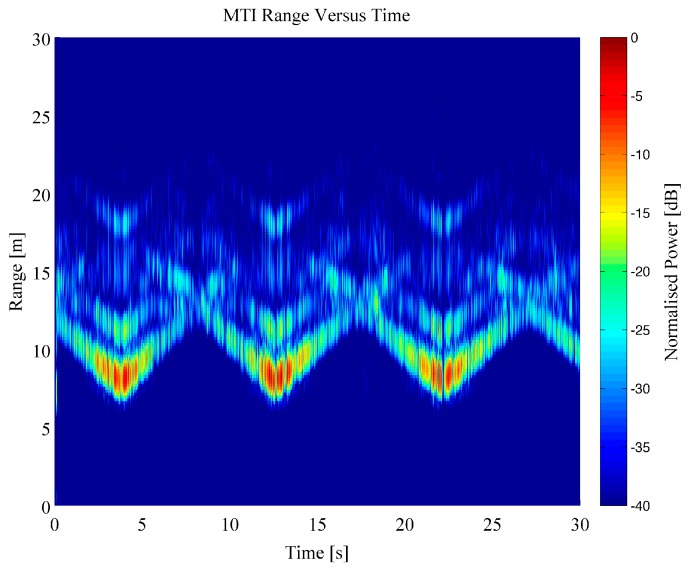
Moving Target Indicator (MTI) plot of individual walking with free hands measured through the wall.

The dataset generated by the Soprano radar system was created by a series of repeat measurements of four separate individuals (labelled A, B, C and D) walking within a closed room in a fixed pattern. The individuals walked up and down within the room directly parallel to the antenna‑pointing direction, following the longest wall in the room. The motion was completed with either no objects being carried or with a rucksack bag being held in both hands which weighed approximately 5 kg. This motion was repeated 93 times in total for all individuals. For each repeat measurement a single recording contained multiple passes of the person walking away and then towards the radar system over a 30 s period. An example Range Time Intensity (RTI) plot of a person walking backwards and forwards within the room can be seen within Figure 5; for this example, the person was walking free handed. The range values include the RF cabling lengths, hence, the target appears at a greater distance than the absolute straight-line distance from the antennas to the individual. The RTI shows that the person was detected within the scene, shown by the disturbance in the signatures at 2.5, 12 and 22.5 s close to the wall signature at 7 m. The RTI image is dominated by the very strong component from the wall reflection as well as the further down range sidelobes of this bright target. It is important to remove this component to enhance the dynamic range of the data dedicated only to moving targets within the scene and this was completed using a Moving Target Indicator (MTI) filter, defined in the previous section. A plot of an MTI processed dataset can be seen in Figure 6, using the same data as shown in Figure 5.

The effect of the MTI filter can be clearly seen in Figure 6, where the moving person target is clearly shown to increase and decrease in range as they walked back and forth within the room. The additional components at longer ranges (greater than 15 m) appear to follow the same slope as the main target and are considered to be generated by multipath due to the complex cluttered scene. The scene used for these experiments was particularly cluttered with lots of opportunity for multipath to be generated. Moreover, the external brick wall which consisted of an air cavity could have potentially contributed a significant amount of multipath, but this represents a real world scenario for the sensor.

**Figure 7:**
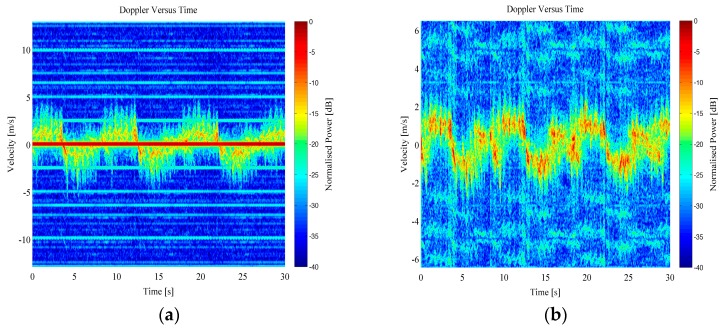
Example Doppler-time spectrogram from a person walking free handed: (**a**) RTI micro-Doppler; (**b**) MTI micro-Doppler.

**Figure 8:**
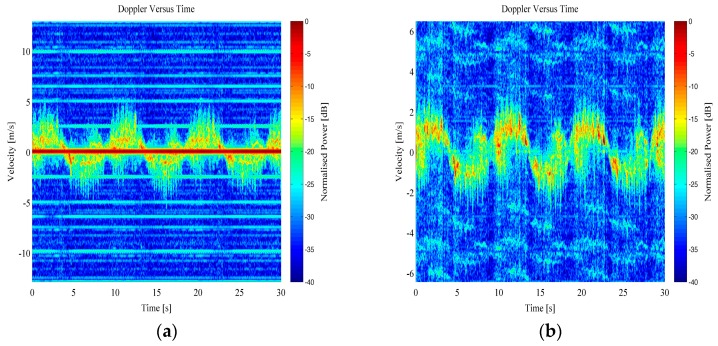
Example Doppler-time spectrogram from a person walking carrying a bag in both hands: (**a**) RTI micro-Doppler; (**b**) MTI micro-Doppler.

The range data was then transformed into the Doppler domain using a Short Time Fourier Transform (STFT) technique. In order to complete this processing, the range bins the target was present within have been selected. A windowed short time FFT processing technique was then applied to these range bins, with a window length of 64 samples and a Hanning weighting. This resulted in a Doppler-time spectrogram that shows both the bulk Doppler component of the person’s velocity while walking away and towards the radar sensor, as well as the micro-Doppler components generated from swinging arms, legs, as well as the torso. Examples of micro-Doppler signatures from both RTI and MTI data can be seen in Figure 7 and Figure 8 for free handed walking and walking whilst carrying a bag in both hands, respectively.

The micro-Doppler data from the MTI signals was then systematically divided into sections of walking towards and walking away from the radar system, which over the full 30 s capture occurred multiple times. The MTI micro-Doppler result was used as it has the dominant zero frequency components removed, which does not contain information useful for motion characterization. The sectioning of the data was completed manually for this analysis, although it could be automated within further developed software. Each of these micro-Doppler sections have been analysed in order to evaluate the SVD components, for both the free handed and carrying case. For a real-time system this methodology would need to be streamlined by an automated computation method but for the offline processing that is being demonstrated within this work, an empirically manual selection was sufficient.

**Figure 9:**
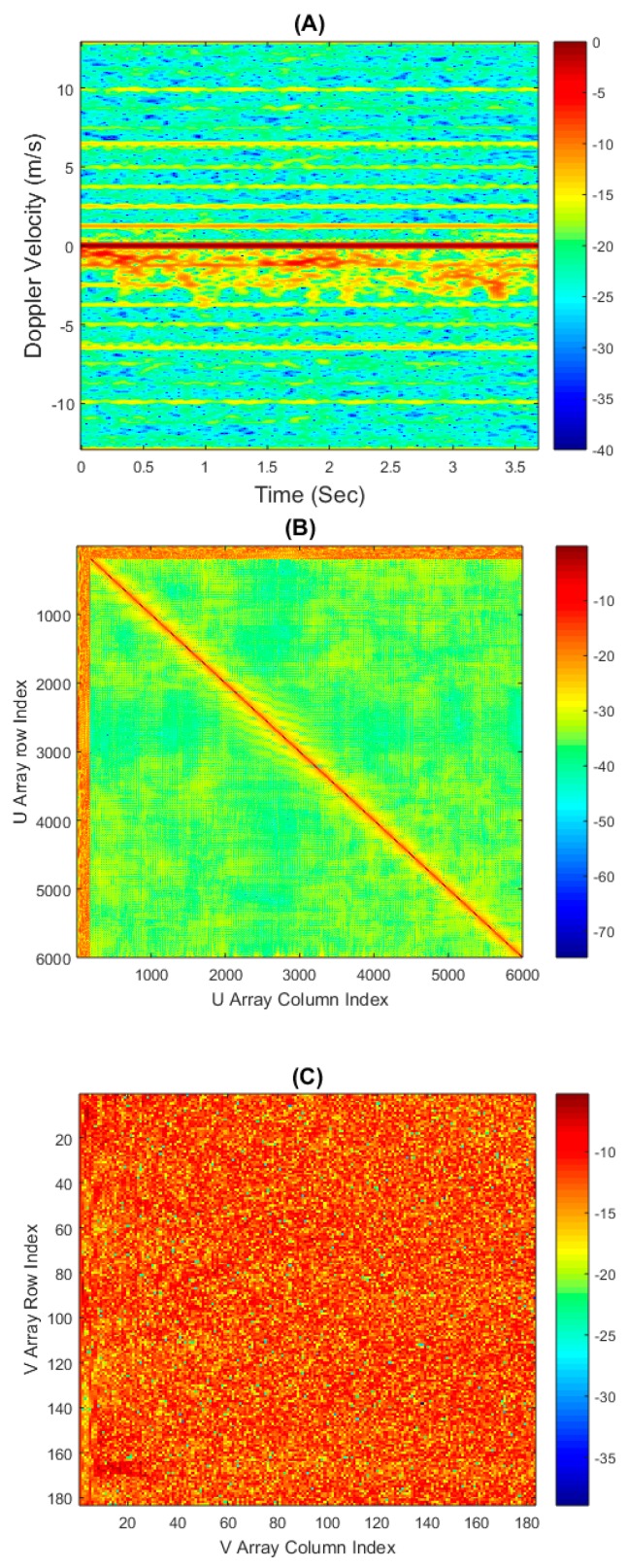
(**A**) micro-Doppler section from free handed motion; (**B**) Singular Value Decomposition (SVD) *u* Array plotted as 10×log10(u) from micro-Doppler section; (**C**) SVD *v* Array plotted as 10×log10(v) from micro-Doppler section.

An example micro-Doppler section, the SVD *u* array and *v* array from a free handed motion can all be seen in Figure 9A–C respectively. The two SVD output matrices are very different in their appearance, they have been plotted as 10×log10(u) and 10×log10(v) to best visually demonstrate the dynamic range of the values obtained. The u matrix has strong components visible at the top and left hand edge, while maintaining a strong diagonal component throughout. The v matrix is more noise like, although at the left hand edge clear non-Gaussian features are visible; hence the feature extraction from this matrix has been isolated to only the first 10 columns of this matrix.

The evaluation of all the SVD matrices from each Doppler section away/towards was completed, and these matrices were then used to extract features that could be input into a classifier to evaluate how easily separable are the two different types of motion. The features that were extracted from the SVD profiles were as follows:

1. Sum of all the values within the first 100 rows within of the u matrix
2. Mean of the diagonal of the u matrix
3. Standard deviation of the diagonal of the u matrix
4. Standard deviation of the first ten columns of the u array
5. Standard deviation of the first ten columns of the v array

**Figure 10:**
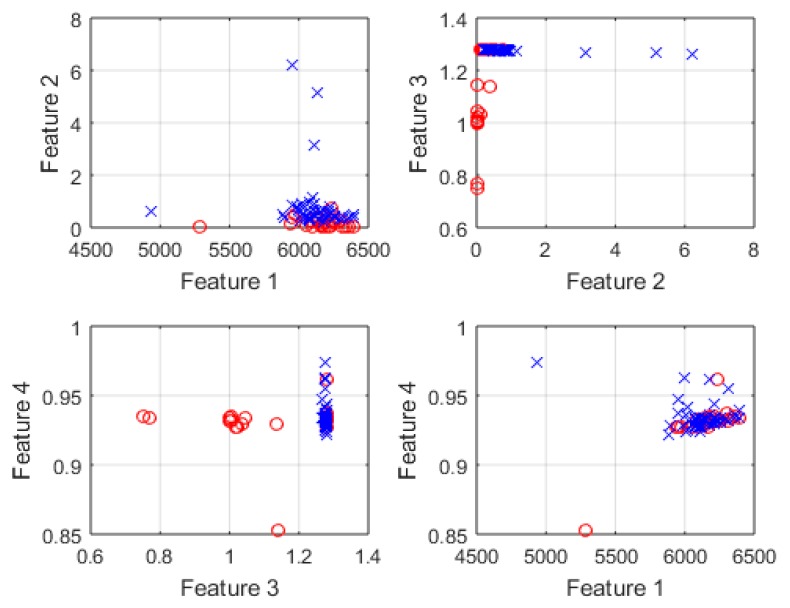
Four example plots of different features plotted against each other. The free hand motion features are shown as blue crosses, while the bag carrying features are shown as red circles.

The numbering used here will continue through the remainder of the paper when referencing each individual feature. Example plots of four different paired combinations of the features listed above can be seen in Figure 10. This qualitatively demonstrates that some feature combinations are much more separable in the feature space than others. The feature pairs that were found to be more separable were features 3 vs. 2 and features 3 vs. 4, whereas features 1 vs. 4 were clearly the least separable.

These obtained features were then used as inputs to a given classifier. These feature have been selected as prior research [31], demonstrated that these values can be successfully used to classify different micro-Doppler motions in non-TTW scenarios. The total number of features extracted for all individuals was 1635 for the free hand walking case and 1470 for the bag carrying case, due to the reduced number of Doppler sections that could be extracted from the bag carrying case.

The first classifier that has been selected for this work was the DA classifier applied in two different variants: the diagonal-linear and diagonal-quadratic. The training stage of the classifier was applied using a randomly selected sub-section percentage of the full dataset features. The classifier was then tested on the remaining samples that were not used in the training process. In order to represent an average training success rate, this process was repeated using 100 Monte-Carlo simulations; all results shown are the average value from these repetitions. The classifier success rate was defined by the output confusion matrix, by summing all correctly indicated classes and dividing by the total number of events, putting no emphasis on either false positive or false negative errors. Different real world scenarios may require emphasis of either avoiding false positives or false negatives, but for this analysis they have been treated equally.

The results from applying the classifier to the separate data from persons A, B, C and D can be seen in Table 2. The training percentage was varied from 20% to 40% in steps of 5% as real classifiers are typically trained with percentages comparable to this. The linear-diagonal DA result was shown to be slightly more effective than the quadratic version averaged across training levels, but by only ~2%. The classifier performance for person C produced the best results, 14% or 16% better than the worst performing result for the linear and quadratic classifier, respectively. This clearly shows that the challenge of assessing if an individual is carrying an item will be strongly dependent on the person being observed; agreeing closely with prior research which has already shown that micro-Doppler signatures can vary significantly between different individuals [45].

**Figure 11:**
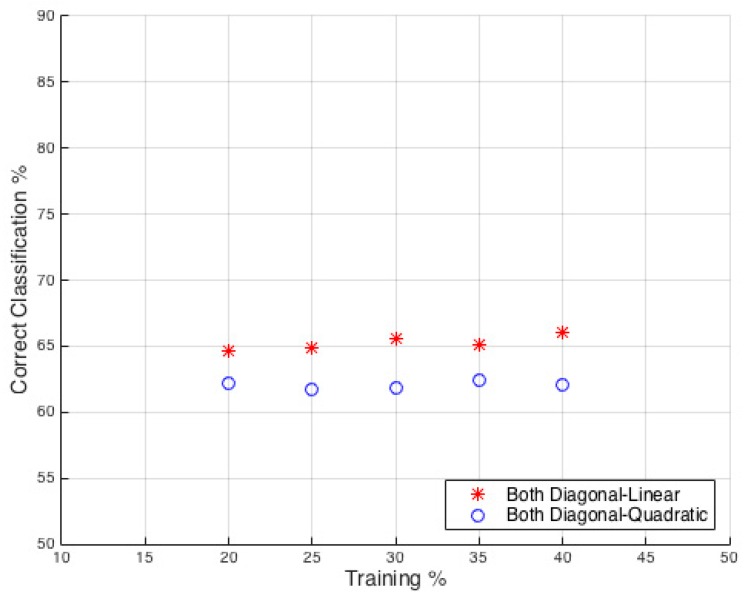
All individuals Data DA Classifier: Classification accuracy plotted against percentage of training set size used.

The DA classifier was then applied to the joint dataset from all individuals to evaluate the overall performance (Figure 11). The results show a classification probability that is approximately an average of the individual results seen in Table 2. Within this result it is worth noting that there is little variation in the performance with increase in train set size, indicating that little additional benefit is gained from training with 20% to 40%. This shows that the previous classifier processing on the individual person data was still statistically significant and not significantly affected by the sample set size.

For comparison the RF classification process was applied to the same data from the four individuals independently, shown in Table 3. These results use all five features for each individual and can be compared directly to Table 2. The RF classifier is shown to be more effective than the DA method, with the greatest difference found for Person D, who showed a 25% increase in classification success rate. On average the RF was found to be 13% better than the DA across all individuals, which is very significant. The trend of classification success against training set size was found to have a greater gradient for the RF method on the joint data compared to the DA classifier. This shows that the RF methodology is more sensitive to the training set size but is able to achieve higher overall classification performance. It was found to benefit by 6% when training with 40% data in comparison to 20%.

**Figure 12:**
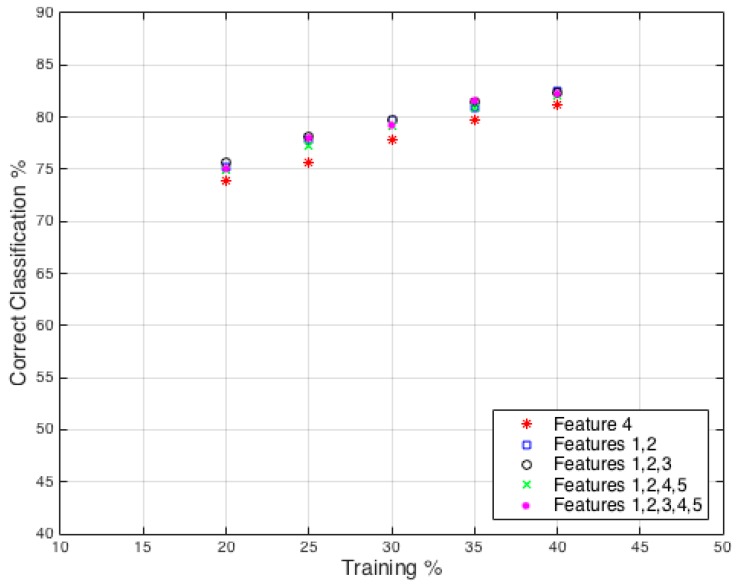
Data from all individuals: Random Forest Classification accuracy plotted against percentage of training set size used.

The classifier results from the full dataset using features from all individuals is shown in Figure 12, which can be compared to the DA results in Figure 11. In this case, the RF classifier was tested using a range of input features from a single feature to all five features. The results shown are the highest percentage achieved by any combination of feature for the defined number of features allowed; the selected features are indicated in the legend. The increase in features used demonstrated diminishing returns or, in fact, reductions in success rates. It was found that higher success rates were achieved when using only three of the five potential features, for example, when training with 30% and 40%. This demonstrates that using more features is not necessarily the best way to approach optimizing this classification problem; effort should be placed on ensuring effective features are selected. On average the RF classifier was found to have 17% and 14% greater classification rate in comparison to the linear and quadratic DA classifiers, respectively. This is a significant difference and shows that SVD classification is possible in a TTW scenario, although the absolute level of success rate is far below an effective reliable solution that could be used in real world scenarios.

**Figure 13:**
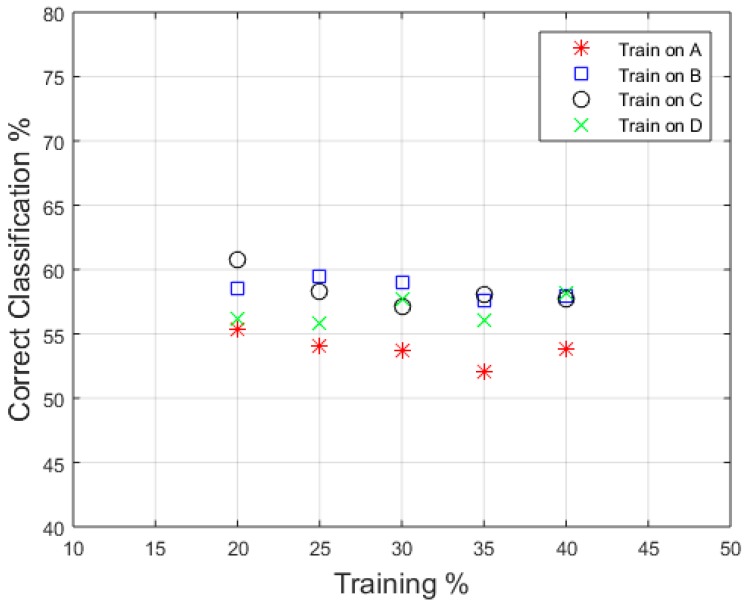
Classification training on single person’s features and then tested on the remaining individuals.

A final test was performed to investigate the effect that training data has on the RF classifier. The previous results show a clear difference in performance based on which individual’s actions are being classified. Therefore, analysis has been completed to evaluate how effective a classifier is when only data from a single person is used to train the classifier. The classifier was then tested on input features from the three remaining individuals. The results from this can be seen in Figure 13, where each different marker represent results from a classifier trained only on a specific individual. For this classification, all five features have been used. A significant reduction was found when compared to the five feature result in Figure 12, showing how person dependent the features extracted are. A focus of future analysis should be on isolating features that are as independent of individual as possible to ensure “blind” classification can be performed effectively on individuals that have not been used as part of the training process.

## 5. Conclusions

The experimental results shown have validated that the 5.8 GHz FMCW system Soprano is capable of detecting human targets through a real brick wall of a house. The MTI processing techniques allowed a significant improvement of SNR and micro-Doppler signatures and are thought to be vital to the pre-processing in order to successfully classify the motions. It was possible to analyse the individual human targets’ micro-Doppler signatures in this TTW configuration through the use of segmentation and feature extraction. The features were extracted using SVD analysis, which has been found to be suitable for TTW micro-Doppler characterization. Components of the SVD *u* and *v* matrices contained vital information regarding how the individual was moving during the measurements. The five SVD features were then successfully used as part of a classification process, by applying simple discriminate analysis and random forest algorithms with varying training set sizes and number of features. The DA and RF classifiers were shown to achieve successful classification rates of up 76% and 94% respectively. The performance of the random forest classifier was found to be more sensitive to training set size, whereas the discriminant analysis method showed little variation across training set sizes of 20% to 40%. The performance varied between each individual’s features, showing that these methods may be linked to how a person moves and for certain people it may be easier to differentiate between carrying and non-carrying situations. The performance of the RF classifier was also assessed when only trained on data from a single person while testing on features from the three other individuals. This was found to reduce the classifier performance by up to 25%, demonstrating how independent each person’s micro-Doppler signature is. These novel results show the potential of micro-Doppler SVD classification in a through wall scenario and confirm the significant potential this has for real-world scenarios. Future analysis will look to expand the overall available dataset to different geometries and wall thickness and to use more individuals to evaluate the resilience of this classification method to changes in these variables.

## Figures and Tables

**Table 1:** Soprano radar parameters.

Parameter	Value
Central Frequency	5.8 GHz
Bandwidth	83.5 MHz
Base-bandwidth	125 kHz
Transmit Power	13 dBm
Noise Figure	2.5 dB
Antenna Beamwidth	30° Azimuth 30° Elevation
Antenna Gain	12 dBi

**Table 2:** Discriminant Analysis (DA) classifier results from each individual person.

Linear						Quadratic				
	Training %					Training %				
Person	20	25	30	35	40	20	25	30	35	40
A	59.7	60.9	60.6	63.6	62.1	58.6	58.0	58.1	59.1	61.3
B	64.6	62.3	63.4	61.9	66.0	59.4	58.4	59.0	58.2	58.7
C	76.2	75.3	77.0	76.3	76.1	74.4	75.7	75.2	75.3	74.3
D	64.0	66.0	65.1	64.9	65.3	65.2	65.5	65.4	66.6	65.1

**Table 3:** Random Forest (RF) Classifier results from each individual person.

Random Forest					
	Training %				
Person	20	25	30	35	40
A	74.7	74.8	73.6	75.3	75.9
B	63.4	65.4	64.1	62.7	65.4
C	80.7	83.6	82.9	84.3	83.2
D	87.8	93.4	92.6	91.2	92.3
